# Effect of Ceramic Scaffold Architectural Parameters on Biological Response

**DOI:** 10.3389/fbioe.2015.00151

**Published:** 2015-10-09

**Authors:** Maria Isabella Gariboldi, Serena M. Best

**Affiliations:** ^1^Department of Materials Science and Metallurgy, Cambridge Centre for Medical Materials, University of Cambridge, Cambridge, UK

**Keywords:** scaffold architecture, ceramic scaffolds, bone tissue engineering, 3D printing, graded materials, scaffold design

## Abstract

Numerous studies have focused on the optimization of ceramic architectures to fulfill a variety of scaffold functional requirements and improve biological response. Conventional fabrication techniques, however, do not allow for the production of geometrically controlled, reproducible structures and often fail to allow the independent variation of individual geometric parameters. Current developments in additive manufacturing technologies suggest that 3D printing will allow a more controlled and systematic exploration of scaffold architectures. This more direct translation of design into structure requires a pipeline for design-driven optimization. A theoretical framework for systematic design and evaluation of architectural parameters on biological response is presented. Four levels of architecture are considered, namely (1) surface topography, (2) pore size and geometry, (3) porous networks, and (4) macroscopic pore arrangement, including the potential for spatially varied architectures. Studies exploring the effect of various parameters within these levels are reviewed. This framework will hopefully allow uncovering of new relationships between architecture and biological response in a more systematic way as well as inform future refinement of fabrication techniques to fulfill architectural necessities with a consideration of biological implications.

## Introduction

Ceramic scaffold architecture has long been explored as a factor to optimize for bone tissue engineering. While architecture has been shown to affect scaffold performance and biological response, a single optimal scaffold architecture does not exist (Bohner et al., 2011). Different functional necessities, such as mechanical performance and permeability, will often require competing properties (Hollister et al., 2002). Optimized structures will also vary according to defect site due to differences in functional requirements and site-specific aspects, such as location of fluid supply (Bohner et al., 2011). Further, while a range of imaging techniques have been adapted to tissue engineering constructs (Vielreicher et al., 2013), designing and characterizing scaffold geometries systematically remain challenging due to limitations of fabrication techniques and the absence of fully descriptive standardized characterization methods (Bohner et al., 2011; Ashworth et al., 2014). Despite these limitations, the authors believe that a controlled study of the effects of architecture at different length scales on biological response would allow developing an integrated model to optimize scaffold architecture based on the requirements of the patient and the specific defect site.

Studies on the effect of scaffold architecture on biological response have been limited by the inability of conventional fabrication techniques, such as gas foaming and porogen leaching, to vary single parameters independently (Bohner and Baumgart, 2004) as well as issues with consistency of the produced structures (Leong et al., 2003). Some improvements to porogen leaching allow independent variation of pore size and interconnections (Descamps et al., 2008). Recent advances in solid freeform fabrication (SFF) have allowed for the production of precise geometries (Chu et al., 2002; Dunlop et al., 2010; Bidan et al., 2012, 2013a), increasing the control on architecture and allowing for the exploration of previously inaccessible geometries. New theoretical frameworks not hindered by the limited capabilities of fabrication techniques are, therefore, needed to design architectures and quantitatively evaluate their performance in terms of specific functional requirements. This systematic evaluation would allow developing a toolkit of architecture-performance relations to tailor scaffold architecture for specific functional specifications.

Discrepancies between *in vitro* and *in vivo* effects of scaffold architecture, for example, due to cell aggregation *in vitro* (Karageorgiou and Kaplan, 2005), have been a challenge in the field. Further, the adaptation of various additive manufacturing techniques for ceramic scaffolds (Leukers et al., 2005; Michna et al., 2005; Seitz et al., 2005), including the use of 3D printing of sacrificial negative molds (Woesz et al., 2005), remains limited by resolution. Features with sizes on the scale of a single cell cannot yet be achieved. However, rapid improvements in resolution of additive manufacturing technologies have occurred for other industrial applications (Chia and Wu, 2015) and their adaptation to the printing of ceramics and other biomaterials is expected to greatly reduce this limitation.

**Figure 1:**
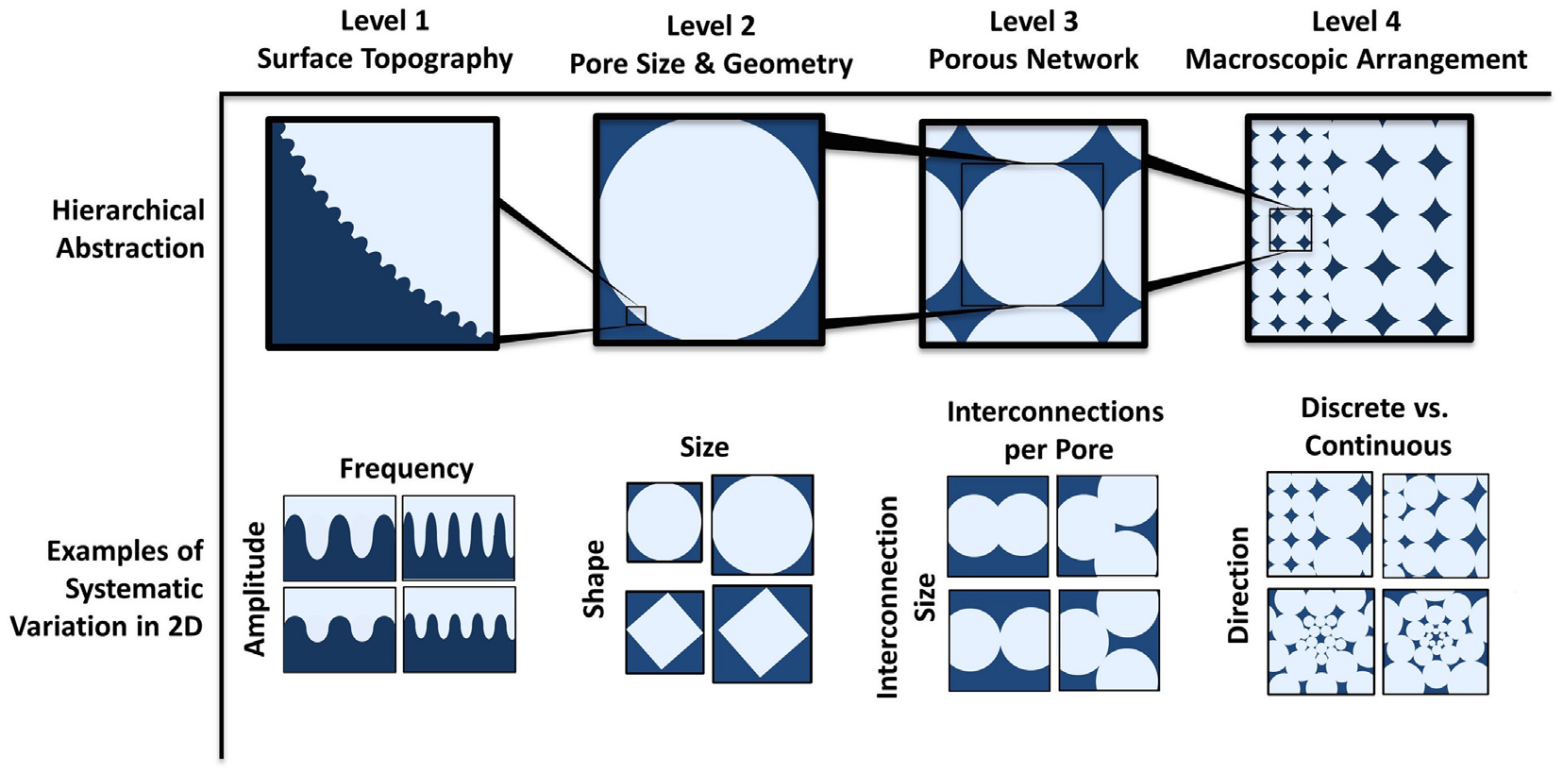
**Theoretical framework for systematic modular design of porous architectures**. This framework consists of four hierarchically scaled levels of abstraction, allowing for independent variation of parameters that give rise to all possible architectures. The levels are respectively the surface topography of the pores that can be sensed by individual cells, the pore size and shape, the interfacing of multiple pores, and the macroscopic organization/variations of pores within the scaffold. Examples of systematic variation in two dimensions within each level are depicted. Examples of parameters that can be varied are amplitude and frequency of the surface roughness profile, the size and shape of the pore, the size and number of interconnections for each pore, and the direction (radial or linear) and profile (discrete change or graded) of spatial variation (of pore size in the pictorial example).

This review aims to develop a new framework for thinking of scaffold architectures and summarize some of the key findings concerning their biological effect (Figure 1). The influence of four levels of architecture, representing different length scales, on biological response will be discussed: (1) surface topography, (2) pore size and geometry, (3) porous networks, and (4) macroscopic pore arrangement.

## Surface Topography

Cells have been shown to sense and react to mechanical cues, such as stiffness (Discher et al., 2005; Engler et al., 2006; Shih et al., 2011), tension (Zhang et al., 2011), and compression (Ramage et al., 2009), through mechanotransduction pathways. A wealth of studies have focused on the effects of surface microtopography on cell response *in vitro* and bone formation *in vivo* with often conflicting results. Microtopography is a poorly defined parameter encompassing features, such as surface roughness and microporosity. Microporosity is commonly defined as the presence of pores with diameters lower than 10 μm (Rosa et al., 2003; Habibovic et al., 2005; Rouahi et al., 2006). Within ceramic struts, micropores can be closed or open (Hing et al., 2005), with closed pores not contributing to the cell microenvironment but affecting the mechanical properties of the struts.

Control over surface roughness and microporosity in bioceramics has been achieved by varying sintering conditions (Bignon et al., 2003; Habibovic et al., 2005), changing processing parameters, such as uniaxial powder pressing load (Rosa et al., 2003) and polishing (Deligianni et al., 2001; Rouahi et al., 2006). Single parameter variations using conventional fabrication techniques, however, remain a challenge. Malmström et al. (2007) produced hydroxyapatite scaffolds by slip casting of 3D-printed sacrificial molds, adding a binder to the slurry to obtain microporosity. This method was proposed to avoid secondary effects that varying microporosity by sintering may have, such as changes in grain size, phase, and chemical composition of the calcium phosphate material.

While conclusions drawn by different studies on the effect of microporosity on bone formation and cell behavior are conflicting, multiple *in vivo* studies have shown positive effects of microporosity in implanted ceramic scaffolds. Comparing identical hydroxyapatite structures differing only by the presence or absence of microporosity demonstrated increased bone ingrowth and bone contact in microporous structures compared to non-microporous hydroxyapatite implants (Malmström et al., 2007). Further, comparing different levels of microporosity has shown that increased levels of microporosity resulted in higher volumes of denser bone at early time points (Hing et al., 2005). A study by Habibovic et al. (2005) on biphasic calcium phosphate (BCP) and hydroxyapatite structures with different levels of microporosity showed that a minimum amount of microporosity is required for osteoinduction.

Various mechanisms have been proposed for microporosity effects on cell and tissue behavior. Microporosity has been proposed to provide anchoring sites for cell extensions (filopodia), thus permitting them to spread and invade the material (Bignon et al., 2003; Annaz et al., 2004). By this mechanism, however, microporosity is not deemed necessary, as demonstrated by ingrowth taking place in low microporosity materials (Bignon et al., 2003) and its effect is seen as important at early time points for initial cell attachment with no notable effect on cell morphology later on (Annaz et al., 2004).

Another proposed mechanism deals with the effect of microtopography on adhesion protein adsorption (Deligianni et al., 2001; Annaz et al., 2004). Deligianni et al. (2001) propose that surface roughness affects the selective adsorption of serum proteins, which in turn affects cell-substrate interaction resulting in improved cell behavior *in vitro* (increasing their adhesion, proliferation, and their detachment strength). Surface roughness was found not to have an effect on alkaline phosphatase (ALP) activity but to delay its expression. Rouahi et al. (2006) have shown a 10-fold increase in protein adsorbed on microporous hydroxyapatite compared to non-microporous hydroxyapatite after 30 min of immersion in complete culture medium. The increased protein adsorption was correlated with a higher initial attachment on microporous hydroxyapatite (within the first 24 h) of osteosarcoma cells (Saos-2), but with a significantly lower proliferation potential after 4 days, possibly due to a closer interaction of cells with the substrate.

Habibovic et al. (2005) propose that increased microporosity and decreased crystal size with lower sintering temperatures increase the specific surface area of the material, favoring the dissolution and reprecipitation of calcium and phosphate ions inducing the formation of biological apatite. Proteins that are co-precipitated in this process induce the differentiation of cells into the osteogenic lineage. Hing et al. (2005) link positive effects of microporosity at early time points after implantation to an increased vascularization of the scaffold, potentially due to increased nutrient permeability or to increased protein adsorption and cell attachment. At later time points, the most significant factor was hypothesized to be strut porosity’s effect on the mechanics of the scaffold with implications for cell mechanotransduction. This proposition was supported by lower total porosity scaffolds resulting in similar bone volume as ones with higher porosity.

Other studies have contradicted these results. An *in vitro* study conducted by Rosa et al. (2003) reported that surface topography of hydroxyapatite did not affect initial stages of cell attachment and that proliferation, protein synthesis, ALP activity, and bone-like nodule formation were increased on surfaces with lower levels of microporosity. Differences in experimental results, particularly *in vitro*, can be ascribed to different experimental design, such as processing techniques, experimental set up, and material chemistry (Malmström et al., 2007). Microtopography and microporosity still remain loosely defined parameters, reported, for example, through roughness values (Deligianni et al., 2001) or as percentages (Hing et al., 2005). New methods of characterizing microtopography with more emphasis on geometric and/or topographical aspects that affect cell contact and morphology could be beneficial. A more systematic characterization is proposed taking into account defined surface profile parameters, such as spatial frequency and amplitude of surface roughness (depicted in level 1 in Figure 1). Further, exploring new scaffold surface topographies could allow new functionality. For example, patterning surfaces with grooves could allow spatially controlling cell orientation by contact guidance (Brunette, 1986; Oakley and Brunette, 1993; Anselme et al., 2002; Chen et al., 2009) within the scaffold, which could allow control over tissue deposition orientation (Wang et al., 2003; Engelmayr et al., 2006).

## Pore Size and Geometry

Macropore size and shape (level 2 in Figure 1) play a key role in tissue formation inside ceramic scaffolds. Karageorgiou and Kaplan (2005) have reviewed the pore size requirements of biomaterials. The minimum pore size requirement for bone ingrowth, however, still remains a highly contested topic. Based on the work of Hulbert et al. (1970), pore size should not be smaller than 75–100 μm. Osteogenesis has been shown by other studies to be enhanced with pore sizes larger than 300 μm (Tsuruga et al., 1997; Kuboki et al., 2001) due to higher permeability and potential for vascularization although higher porosity results in diminished mechanical strength. However, multiple recent studies have shown both bone ingrowth and the presence of cells in micropores (Lan Levengood et al., 2010; Bernstein et al., 2013; Polak et al., 2013). Regardless, pore shapes produced by conventional fabrication techniques being largely irregular results in difficulty in defining pore size. Pore size can be analyzed using different quantitative analytical techniques, such as mercury intrusion porosimetry or imaging techniques including X-ray microtomography (Atwood et al., 2004; Jones et al., 2007) combined with various analytical methods. A single pore size value or pore size distribution is insufficient for describing a porous architecture. Results greatly differ depending on the analytical model used, such as a continuous or discrete approach, for the analysis of pore size distributions (Münch and Holzer, 2008). Further, incongruence in analytical approaches results in discrepancies in the literature and generates results that cannot be compared to yield definitive conclusions.

**Figure 2:**
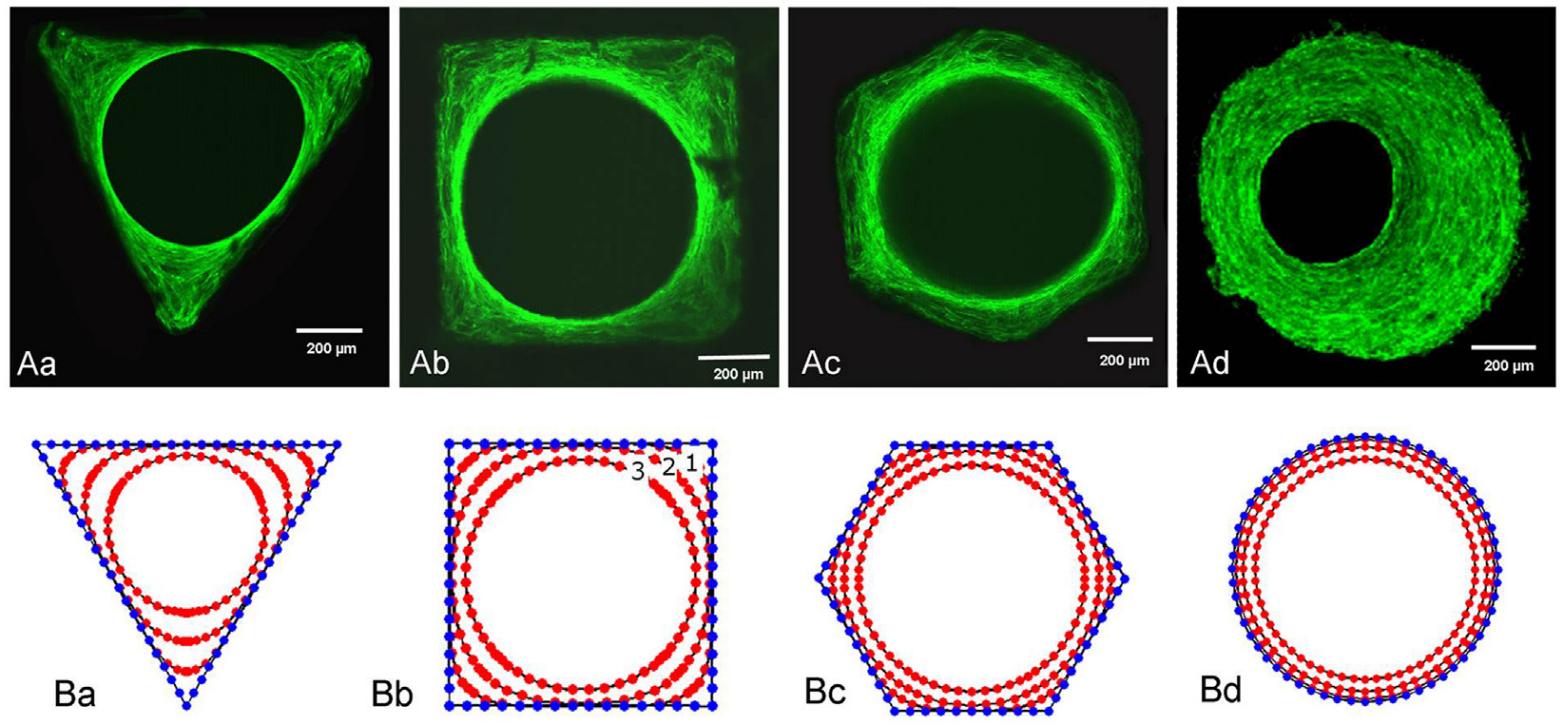
**Tissue growth in channels with controlled geometry**. Tissue growth in channels with (i) triangular, (ii) square, (iii) hexagonal, and (iv) circular cross-sections. **(A)** Actin stress fibers stained with phalloidin-FITC after 21 days for (i)–(iii) and 30 days for (iv). **(B)** Computational simulation of tissue growth showing evolution of tissue front at different time points. Computational results closely agree with experimental results. Figure retrieved from Rumpler et al. (2008).

Macropore geometry has been found to modulate cell-network formation and tissue growth. A thorough review on this topic has been carried out by Zadpoor (2014). Cells have been found to respond to radii of curvature far larger than the cells themselves (Rumpler et al., 2008). Murine osteoblast-like cells cultured on hydroxyapatite channels with controlled cross-sections of different geometries (triangular, square, hexagonal, and circular) were shown to have initial tissue formation occur at corners, with cells on edges not growing until growth of adjacent tissue resulted in a curved environment (Rumpler et al., 2008) (Figure 2). Tissue growth is proposed to be curvature-driven, with growth increasing with local curvature resulting in a round opening regardless of the initial substrate shape. High curvature is thought to result in mechanical forces in cells, as evident by the formation of actin stress fibers along the tissue-fluid interface, which drives further tissue growth (Nelson et al., 2005). Overall tissue growth was independent of shape, but dependent on the cross-sectional perimeter (or channel surface area), with shorter perimeters resulting in more tissue at any given time point, consistent with Fenchel’s theorem (Fenchel, 1929) that states that the average curvature of a closed convex plane is only dependent on perimeter. The model was confirmed to accurately predict the change in curvature profiles and the amount of tissue produced in pores with more complex geometries (Bidan et al., 2013a), with both experimental and computational results showing that at early time points, growth rates for cross shaped pores are almost twice those of square-shaped pores regardless of their size.

Observation of tissue growth on physiologically relevant geometries has shown that the curvature-driven growth model likely regulates bone architecture emerging after bone remodeling. Tissue growth in circular pores, simulating cavities preceding osteon formation, proceeds with a concentric tissue front, while growth in semi-circular trenches, mimicking ridges preceding hemi-osteon formation, results in a pinned tissue front and an eventual termination of growth upon flattening out (zero curvature) (Bidan et al., 2012). This cell-network behavior is consistent with physiological observations of trabecular bone’s three-dimensional curvature approaching zero (Jinnai et al., 2002; Bidan et al., 2012).

Various models have been developed to predict tissue growth in geometrically controlled environments. Dunlop et al. (2010) proposed a thermodynamically based model for tissue growth that successfully predicted experimental results by Rumpler et al. (2008) based on the theoretical models previously developed by Ambrosi and Guana (2005) and Ambrosi and Guillou (2007) on stress-modulated tissue growth and the biochemical energy in tissue growth respectively. Bidan et al. (2013a) developed a mathematical model to describe total tissue growth rate in a scaffold. The model includes factors representing cell activity, scaffold properties, and pore structure, including geometry. The model was extended to structures containing pore geometry heterogeneity.

**Figure 3:**
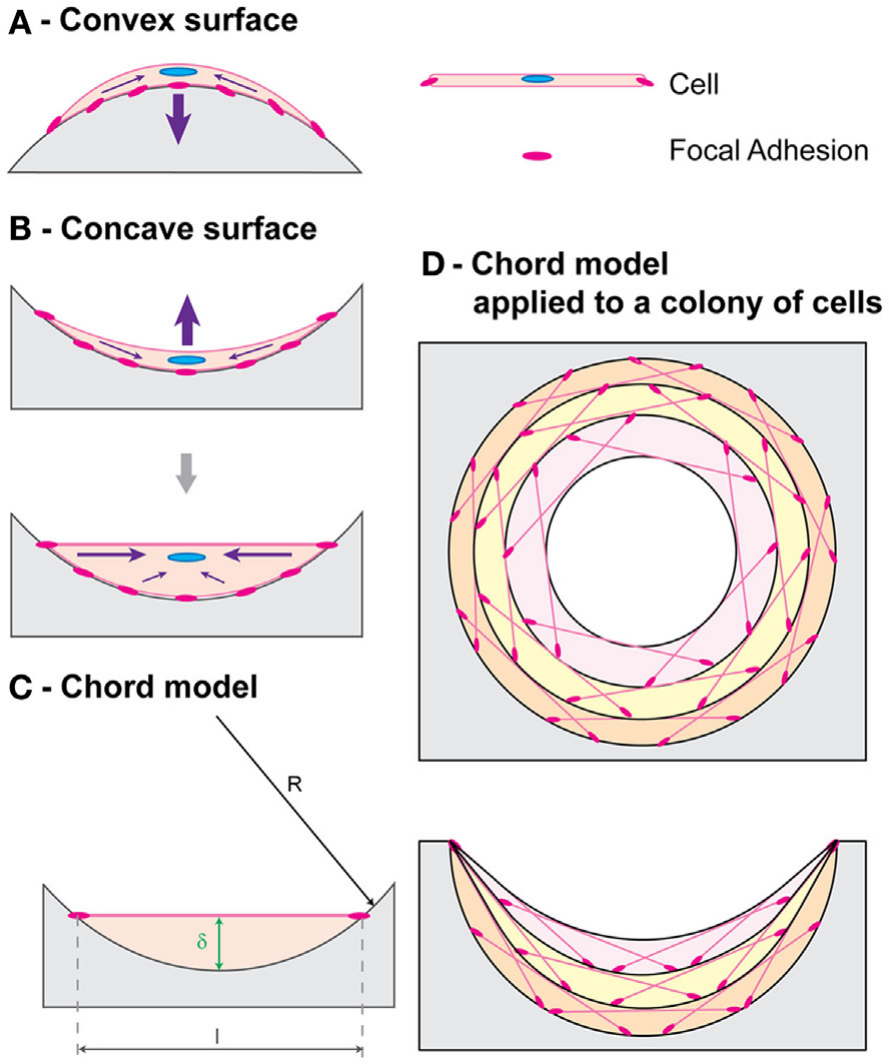
**Chord model**. A simple geometric framework for curvature-driven growth developed by Bidan et al. (2012) is shown. Cell contraction (purple arrows) resulting in stable tensile state morphology is depicted for **(A)** a convex surface and **(B)** a concave surface. **(C)** A cell with thickness δ proportional to the local curvature is represented with a chord. **(D)** Chord segments can be combined to represent tissue growth, with each layer acting as a substrate for the one above. Figure retrieved from Bidan et al. (2012).

Improvements to the curvature-driven growth model have included its refinement to explain differences in tissue growth on concave versus convex surfaces (Gamsjäger et al., 2013) and its extension to predict growth in three dimensions (Bidan et al., 2013b; Guyot et al., 2014). Tissue growth has been experimentally observed *in vivo* to be considerably increased on concave surfaces compared to convex and planar ones, with bone formation initiation occurring at concavities in hydroxyapatite-coated titanium implants (Ripamonti et al., 2012; Scarano et al., 2014). Gamsjäger et al. (2013) incorporate the role of surface stress in the curvature-driven growth model to allow predicting this phenomenon. An explanation for this behavior is the tensile nature of the cells that make up the tissue surface, as evidenced by actin and myosin presence, that affects the growth of the cells underneath the interface differently depending on the nature of the underlying surface, as explained by the chord model developed by Bidan et al. (2012) (Figure 3).

**Figure 4:**
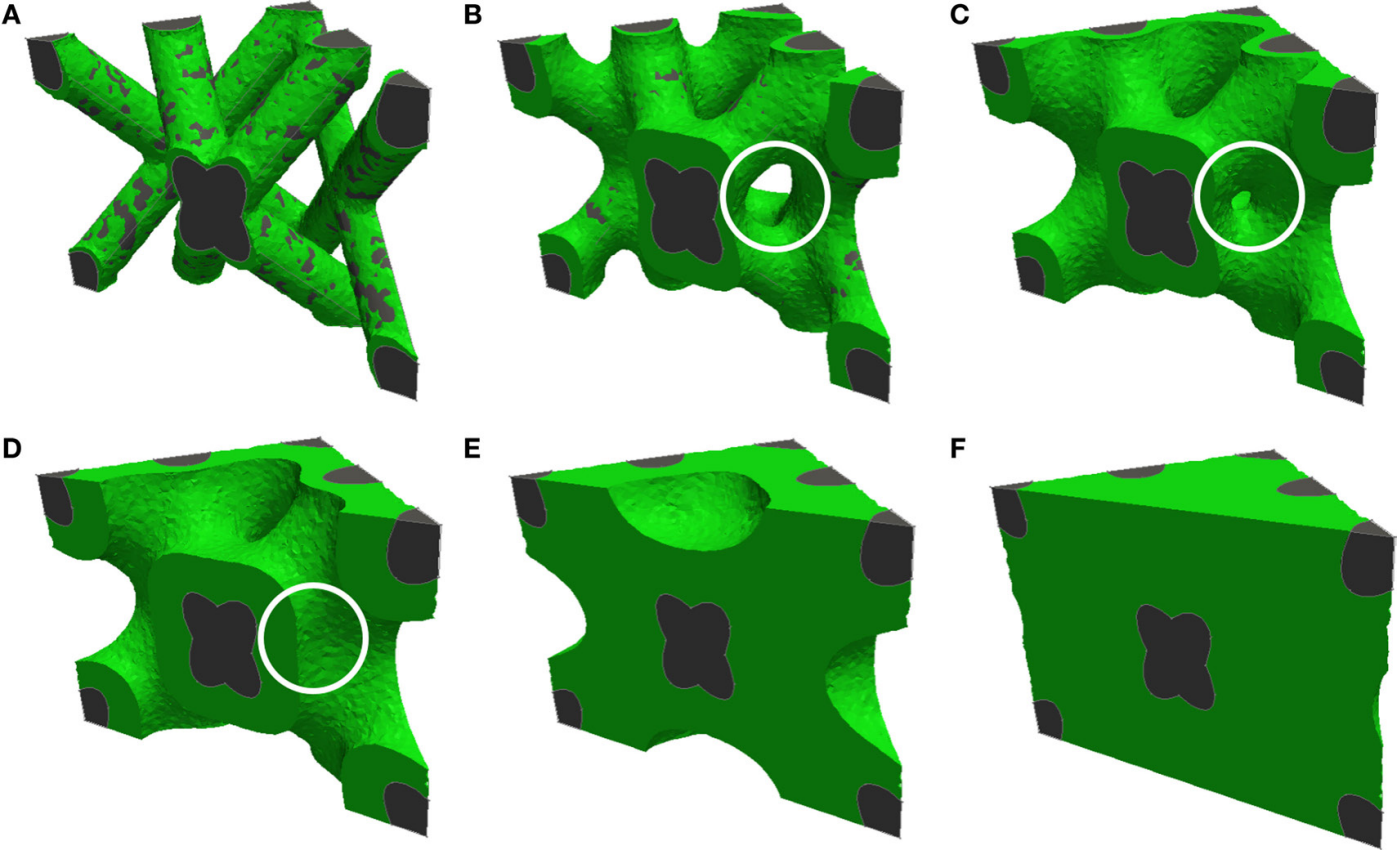
**Computational prediction of tissue growth on a complex 3-dimensional pore geometry**. Cross-sections of diamond-shaped pores are shown, with increasing degrees of pore filling from **(A)** to **(F)**. The white circle highlights bridging of tissue resulting from convergent growth from two local surfaces [reproduced from Figure 7 from Guyot et al. (2014) with kind permission from Springer Science and Business Media].

Computational models have been developed for predicting curvature-driven growth of 3-dimensional geometries (Bidan et al., 2013b; Guyot et al., 2014). Guyot et al. (2014) developed a model that can be applied to highly complex and non-symmetrical scaffold geometries with minimal user input (Figure 4) and was validated *in vitro* on Ti6Al4V (Ti) scaffolds produced by selective laser melting. All simulations were found to eventually yield spherical or cylindrical tissue fronts. The authors of the study proposed that given scaling differences in experimental versus simulation times with pore size, integrating in the model the effects of mass transport and other mechanisms necessary for biological function may improve its predictive ability.

Pore geometry could also have large implications in regulating collagen fiber organization and orientation, with profound effects on the resulting tissue structure and mechanics (Engelmayr et al., 2006). Tissue growth in open rectangular pore slots with different widths in calcium phosphate bone cement plates showed that actin fibers were organized parallel to the pore length in slots of 200 and 300 μm thickness. Larger slots were found to have fibers forming larger angles with the longitudinal axis at the growth front and thinner fibers oriented mainly normal to the pore wall in the bulk of the tissue (Knychala et al., 2013). The anisotropy created by the actin alignment was proposed to be encouraging tissue growth by migration and force transmission through cell–cell junctions (Tambe et al., 2011).

Given the findings of geometric effects on tissue growth, it is clear that additive manufacturing techniques currently being adapted for biomaterial scaffold fabrication are often inadequate. Many extrusion-based techniques, for example, produce arrays of cylindrical rods (Michna et al., 2005; Seitz et al., 2005; Carrel et al., 2014) that are inadequate given their convex geometry. Biological effects must be taken into consideration for further refinement of SFF methods for scaffold fabrication.

## Porous Network

The interfacing of two or more pores to form a network (level 3 in Figure 1) affects the infiltration of nutrients and cells into the scaffold. The macroscopic structure produced by a network of pores is often described using porosity values. Overall scaffold porous structure is a key determinant of scaffold mechanical performance (Rodríguez-Lorenzo et al., 2002), resorption rate (De Groot, 1988; Bohner and Baumgart, 2004) as well as the surface area to volume ratio (SAV) of the scaffold (Ashworth et al., 2014). Scaffold SAV has been shown to influence the SAV of the formed tissue in silk fibroin implants (Hofmann et al., 2007). Porosity can be open, closed, or blind-ended, with only open porosity being directly conducive to tissue ingrowth (Ashworth et al., 2014).

Porosity alone, however, while widely used is a poor predictor of biological response. Other than pore size, the arrangement of two or more pores in space to form a porous network requires careful consideration of pore interconnection size and geometry that will affect overall scaffold permeability and accessibility to cells and nutrients. The shape of interconnections is likely an important factor given the curvature-driven growth model and the convex nature of interconnections in many traditional porous scaffolds although this aspect has not been systematically explored to the authors’ knowledge. Two main factors affecting the accessibility of a scaffold to tissue growth are discussed: interconnectivity, which determines the accessibility of a porous network through fenestrations between pores to a finitely sized object, such as a cell, and tortuosity, which determines how long and winding a pathway of interconnected pores is.

Interconnectivity is often considered a binary property, with scaffolds frequently being described as “fully interconnected.” This definition, however, fails to recognize the importance of interconnection size. Cells have been shown to penetrate porous networks with interconnections smaller than the cells themselves (Polak et al., 2013), with deformation of the nucleus being considered a major limitation to cell migration (Wolf et al., 2013). Interconnection size does, however, affect scaffold accessibility to cells. Fenestration size distribution or average interconnection sizes alone are not sufficiently informative parameters as a single small interconnection is enough to prevent accessibility to all downstream pores. Otsuki et al. (2006) showed that narrow pore throats, particularly in the shorter routes connecting a given pore to an implant’s outer surface, compromise tissue differentiation in that pore. They proposed two new indices to assess the effect of narrow pore throats on the entire implant’s performance: the volume ratio of dead pores (those pores that do not connect to the implant’s outer surface due to narrow pore throats) and the average detour index (the ratio of the shortest distance through interconnections of all pore voxels, excluding dead pores, to the distance from the implant periphery). Both indices are a function of the minimum pore throat size for bone ingrowth, a value that is dependent on implant properties, such as material type.

**Figure 5:**
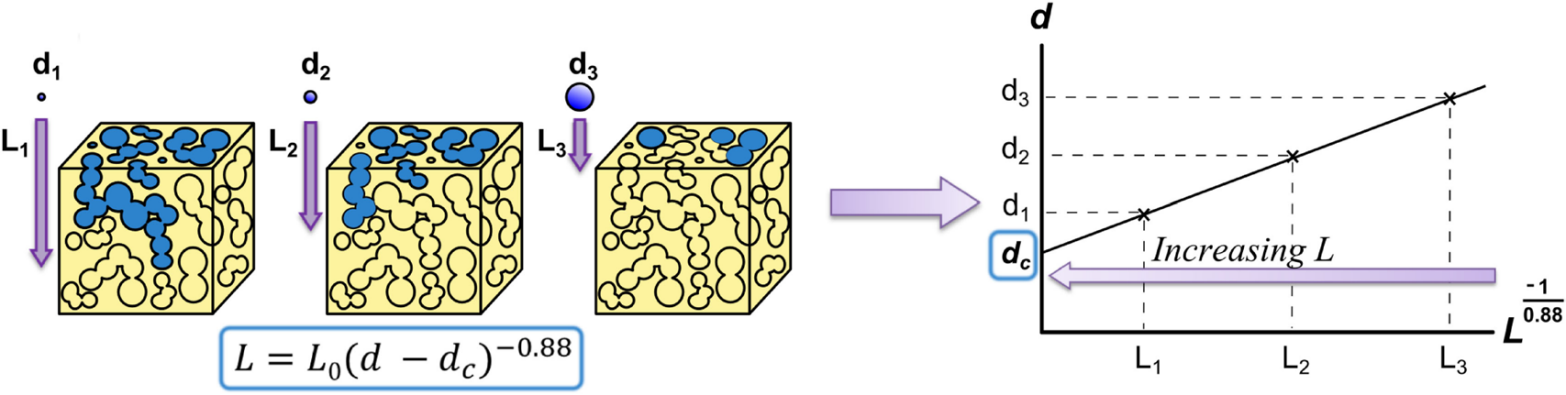
**Method for calculating percolation diameter presented by (Ashworth et al., 2014, 2015) based on a relationship from percolation theory (Saxton 2010)**. The maximum distance traveled in the *z*-direction (*L*) for spheres with different diameters (*d*) is plotted, allowing to infer the percolation diameter, *d*_c_, the diameter of the largest sphere that can percolate through an infinitely long scaffold. The value 0.88 is the percolation constant for 3D systems (Sotta and Long, 2003). Figure adapted from (Ashworth et al., 2015).

Ashworth et al. (2014) developed another solution to the problem of quantitatively describing an entire scaffold’s interconnectivity by adapting the concept of percolation from geological research to describe the interconnectivity of porous networks. A percolating cluster of pores is defined as a cluster of interconnected pores that forms a path through the material. Ashworth et al. (2014) introduce the new scalable quantity of percolation diameter, i.e., the diameter of the largest “tracer” sphere that can percolate through an infinitely large porous structure (Figure 5).

While a percolation diameter allows the determination of whether an object with a specific size is able to fully traverse a scaffold, tortuosity describes how circuitous a route through a network of pores is. Tortuosity is commonly defined as the ratio of the path length through interconnected pores between two points to the length or shortest distance between them (Starly et al., 2007; Chang and Wang, 2011). Another commonly used definition for tortuosity is in terms of the ratio of the diffusivity of molecules in the bulk to the effective diffusivity (Hrabe et al., 2004; Zalc et al., 2004). This second macroscopic definition, however, is only informative for symmetrical geometries (Starly et al., 2007). Tortuosity affects cell migration through the scaffold, nutrient diffusion, and waste removal. Since tortuosity affects permeability to nutrients necessary for cell proliferation, directed cell growth can be obtained by controlling tortuosity in different directions (Starly et al., 2007). Malda et al. (2004) studied the effect of scaffold architecture on oxygen supply, cell distribution, and cartilaginous matrix deposition. Botchwey et al. (2003) showed that tortuosity affects fluid and nutrient perfusion in scaffolds using dynamic culture conditions, with increased tortuosity resulting in decreased internal fluid flow rates. Random open porosity has also been shown to decrease cell penetration into the hydroxyapatite scaffold core when compared to open porous scaffolds with aligned channels both *in vivo* and *in vitro*, suggesting that tortuosity reduces cell penetration (Silva et al., 2006). On the other hand, tortuous channels have been proposed to increase the rate of osteoblast precursor growth (Leber et al., 2010). Starly et al. (2007) developed a tracer metric numerical model to obtain three tortuosity factors to describe tortuosity for each axis of a unit cell of the scaffold. The model is applicable to any geometry provided that a virtual model can be produced, for example, by use of X-ray microtomography. Tortuosity could also be measured as a function of the size of an object traveling through the scaffold, as is done with a percolation diameter, by using a “tracer” with finite dimension, resulting in a parameter similar to the average detour index proposed by Otsuki et al. (2006) (above).

## Macroscopic Pore Arrangement

Porous scaffolds produced by conventional fabrication techniques are largely homogenous. Novel production techniques, such as SFF and freeze-casting, to some extent, have allowed for the production of porous networks with spatial control over architecture (Hutmacher, 2001) (level 4 in Figure 1). Macroscopic control of porous networks can allow locally tuning the scaffold properties and directing biological activity. This can be done, for example, by spatially controlling mechanical properties, tuning degradation behavior, or directing fluid and nutrient flow. Three different ways of macroscopically controlling scaffold architectural properties are discussed: pore network orientation, architectural gradients, and patterning.

Both macroscopic architectural features (such as osteonal and trabecule orientation) and microscopic ones (such as collagen and lamellar orientation) contribute to bone anisotropy (Majumdar et al., 1998; Takano et al., 1999; Doblaré et al., 2004). Bone anisotropy is both necessary for biomechanical function and maintained through bone turnover thanks to cell mechanosensitivity guiding bone remodeling. This results in osteons produced by the cutting cone resorption process being aligned with the major stress axis (Lanyon and Bourn, 1979; Burr and Martin, 1989; Petrtyl et al., 1996). Isotropic pore structures in biomaterials result in tissue that differs from native tissue structurally and functionally (De Mulder et al., 2009). Thus, in order to regenerate anisotropic tissue, anisotropy must be incorporated in the scaffold porous structure (Engelmayr et al., 2008), reducing the need for a second remodeling step (De Mulder et al., 2009). Other than to tune mechanical performance, porous structure anisotropy can be used to guide tissue growth and nutrient flow direction. Optimal pore orientation, therefore, requires defect site-specific considerations to tailor the structure, for example, to the location and direction of fluid supply (Bohner et al., 2011).

Multiple studies have produced anisotropic structures, for example, through ionotropic gelation of alginate-hydroxyapatite slurries (Despang and Bernhardt, 2013) or unidirectional freezing producing columnar or lamellar microstructures (Fu et al., 2009). Ice-templating of scaffolds is a promising technique for creating oriented porous structures by controlling thermal gradients (Pawelec et al., 2013). While pore geometry control is limited, macroscopic control of architectures can be achieved by changing the freeze-drying experimental set up (Moon et al., 2003; Munch et al., 2009), using additives (Munch et al., 2009) and changing thermal profiles (freezing protocols) (Pawelec et al., 2014). Radial pore geometries have been produced by freeze-casting using metallic cylinders with a Teflon rod in the center (Moon et al., 2003).

Chu et al. (2002) have used 3D-printed sacrificial molds to compare orthogonal versus radial channel designs, showing that regenerated bone tissue morphology can be tuned by varying channel design/orientation. However, no studies to the authors’ knowledge have systematically showed the advantages of anisotropic pore structures over isotropic pore structures, controlling factors, such as overall porosity and mechanical properties. Pore anisotropy is also likely to affect the preferred direction of cell invasion and the force transmission to cells *in vivo*. A thorough evaluation of pore structure anisotropy under mechanical loading could allow a better understanding of the benefits of anisotropic scaffolds. Further, Lu et al. (1999) have introduced the concept of “interconnection density” as a factor affecting cell penetration and bone formation. Pore anisotropy would result in differing interconnection densities depending on the cross section, suggesting that oriented porosity could be used to promote directional tissue growth using pore orientation.

Spatially grading porosity is an effective way of locally varying properties, such as mechanical strength and permeability. Simske et al. (1997) describe four length scales of porosity performing different useful functions ranging from enhancing bone ingrowth to fixation during surgery. Different tissues and cell types favor different pore sizes depending on the scaffold material (Oh et al., 2007), which motivates tuning porosity both to allow to tailor for different cell types that might exist in a tissue and to allow for different tissue interfaces, such as between cartilage and bone or blood vessels and bone. Further, graded biomaterials have been shown to allow for the formation of cell density gradients and extra-cellular matrix gradients within the material, allowing for localized control of tissue formation and properties (Woodfield et al., 2005). A review by Miao and Sun (2010) on graded/gradient biomaterials is recommended for a detailed description of fabrication techniques that have been used to achieve pore gradients.

Possibly, the most common approach to spatially graded porous ceramic materials has been the biomimetic design of scaffolds with a denser outer shell mimicking cortical bone and a porous core-simulating trabecular bone. Tampieri et al. (2001) achieved this through multi-step dipping of cellulosic sponges in slurries with different hydroxyapatite crystallinity. Werner et al. (2002) have used tape casting of hydroxyapatite slurries with differently sized porogens. Fierz et al. (2008) used 3D printing of nanoporous hydroxyapatite to create biomimetic structures with large central channels to enhance bone ingrowth in the center of the scaffold. While the biomimetic argument has largely motivated scaffold designs, again, a systematic approach to judge the improved performance of biomimetic structures and graded porous structures has not been performed to the authors’ knowledge.

Last, spatial patterning of scaffold architecture could give rise to localized tuning of scaffold properties. Silva et al. (2006) produced hydroxyapatite scaffolds with open random porosity with and without anisotropic channels. The purpose of the anisotropic macro-architecture was to avoid necrotic core formation by preventing newly formed tissue blocking nutrient exchange with the core of the scaffold (Ishaug et al., 1997). Patterning the scaffold with channels was found to both enhance cell infiltration of the scaffold as well as direct tissue growth.

Scaffolds can be patterned in a variety of ways to spatially control structure and function. Architecture could be used to tune mechanical properties of the scaffold and also the mechanical environment of the cell. The cutting cone resorption process of bone carried out by groups of cells referred to as basic multicellular units (BMUs) is mechanically guided (Van Oers et al., 2008). This results in the direction of osteons having been found to match the major stress axis (Lanyon and Bourn, 1979; Burr and Martin, 1989; Petrtyl et al., 1996). One can imagine a scaffold architecture where tubular pores are spatially graded to have an orientation dependent on the local major stress axis such that newly formed bone has a morphology suited to the mechanical environment.

Roughness has been found to have profound effects on cell behavior, as previously mentioned, with high roughness resulting in strong cell attachments but low proliferation rates (Rouahi et al., 2006). Spatially grading roughness, for example, by use of additives (Malmström et al., 2007), could potentially enhance scaffold performance. One could imagine a structure with varying roughness to encourage proliferation and migration or attachment at different parts of the scaffold. An example of this would be using high roughness outside the scaffold to encourage integration but lower roughness at pore inlets to encourage cell migration toward the core of the scaffold.

Last, spatial control of architecture could allow controlling degradation rate and tissue formation. For example, SAV could be varied by controlling pore size and surface roughness while maintaining a mechanically appropriate local porosity. This would allow controlling degradation rates throughout the scaffold. Pore size and shape could also be tuned spatially to control locally cell type infiltration, whereas macroscopic structures and pore orientation could be used to direct the diffusion of nutrients within the structure.

## Conclusion

The advent and continual development of technologies, such as additive manufacturing that allow for a high degree of control over scaffold geometry, serve the basis for the exploration of precise geometries on cell and tissue behavior. A theoretical framework for systematic design of scaffold architecture at four different levels has been proposed. This framework will hopefully encourage the development of a suite of parameters to describe architecture that will allow a systematic design and evaluation of architectures quantitatively. Novel fabrication methods have also allowed for the development of architecturally heterogeneous materials. Evaluating the performance of architectures on these four levels in terms of outputs, such as mechanical performance, permeability, degradation behavior, cell response, and tissue microarchitecture, will assist in developing a structure–function relationship toolkit that could help develop biomaterials with locally optimized architecture in a way that is patient and defect-site specific. Further, the uncovering of these relationships will allow tuning fabrication techniques to architectural necessities rather than adapting technologies developed with insufficient consideration of biological implications.

## Conflict of Interest Statement

The authors declare that the research was conducted in the absence of any commercial or financial relationships that could be construed as a potential conflict of interest.
